# ‘*We can do only what we have the means for*’ general practitioners’ views of primary care for older people with complex health problems

**DOI:** 10.1186/s12875-015-0249-2

**Published:** 2015-03-14

**Authors:** Anna Herzog, Beate Gaertner, Christa Scheidt-Nave, Martin Holzhausen

**Affiliations:** Department of Biometry and Clinical Epidemiology, Charité Universitätsmedizin, Berlin, Germany; Department of Epidemiology and Health Monitoring, Robert Koch Institute, Berlin, Germany; Alice Salomon University of Applied Sciences, Berlin, Germany

**Keywords:** Primary health care, General practitioners, Qualitative study, Attitude, Geriatric health services, Comprehensive health care

## Abstract

**Background:**

Due to demographic change, general practitioners (GPs) are increasingly required to care for older people with complex health problems. Little is known about the subjective appraisals of GPs concerning the demanded changes. Our objective is to explore how general practitioners view their professional mandates and capacities to provide comprehensive care for older people with complex health problems. Do geriatric training or experience influence viewpoints? Can barriers for the implementation of changes in primary care for older people with complex health problems be detected?

**Methods:**

Preceding a controlled intervention study on case management for older patients in the primary care setting (OMAHA II), this qualitative study included 10 GPs with differing degrees of geriatric qualification. Semi structured interviews were conducted and audio-taped. Full interview transcripts were analyzed starting with open coding on a case basis and case descriptions. The emerging thematic structure was enriched with comparative dimensions through reiterated inter-case comparison and developed into a multidimensional typology of views.

**Results:**

Based on the themes emerging from the data and their presentation by the interviewed general practitioners we could identify three different types of views on primary care for older people with complex health problems: ‘maneuvering along competence limits’, ‘Herculean task’, and ‘cooperation and networking’. The types of views differ in regard to role-perception, perception of their own professional domain, and action patterns in regard to cooperation. One type shows strong correspondence with a geriatrician. Across all groups, there is a shared concern with the availability of sufficient resources to meet the challenges of primary care for older people with complex health problems.

**Conclusions:**

Limited financial resources, lack of cooperational networks, and attitudes appear to be barriers to assuring better primary care for older people with complex health problems. To overcome these barriers, geriatric training is likely to have a positive impact but needs to be supplemented by regulations regarding reimbursement. Most of all, general practitioners’ care for older people with complex health problems needs a conceptual framework that provides guidance regarding their specific role and contribution and assisting networks. For example, it is essential that general practice guidelines become more explicit with respect to managing older people with complex health problems.

## Background

In recent years, demographic changes in the constitution of general practitioners’ (GP) clientele have demanded alterations in ambulatory clinical practice to adequately meet older patients’ health care needs [1]. Older people with complex health problems are characterized by multiple, simultaneous, and synergetic health problems, functional limitations, and psychosocial challenges [2,3]. The term ‘complex health problems’ refers to any health problems that require a multidimensional and personalized response from primary care services. This includes combinations of concurrent diseases (multimorbidity) as well as any combination of functional health problems compromising the ability to perform core activities of daily life. Appropriate response may involve the provision of medical care as well as aligned social services. The treatment and care of older people with complex health problems thus do not primarily focus on the cure of single identified diseases but rather on syndromes and interdependent multimorbidity, as well as patient complexity that is the influence of not only health-related characteristics but also cultural, socioeconomic, environmental, and patient behavior characteristics [4]. On the basis of the target group's needs, it is argued that the provision of health care for older people requires integrated networks of health care and social services to provide for continuity of care [5].

Primary care for older people with complex health problems poses a significant challenge to a GP’s geriatric knowledge and ability to synergize various types of information and could best be tackled in a multiprofessional team [6]. In Germany, multiprofessional teams are not the norm and specialized geriatric care has so far largely been restricted to the hospital setting. Usually, the general practitioner will see all patients and refer patients to specialists and therapists depending on his or her clinical evaluation. In Germany, as in other countries, the level of geriatric training varies highly among GPs [7,8].

Few studies have been undertaken to elucidate the professional action patterns and attitudes of GPs regarding geriatric health-care. Some findings suggest that the level of specialized professional training can play a role in the utilization of specific assessments and recognition of responsibilities [9-11]. Additionally, attitudes and perspectives of GPs will influence the range of health problems encompassed in their subjective expertise [9] and their treatment decisions [12]. The separation among professional domains is repeatedly highlighted to be a barrier, and interprofessional cooperation is shown to be a chance for comprehensive treatment and care in older patients [1,9,13,14].

A number of recent studies are available that address GPs’ attitudes and practices concerning the concept of age and aged patients [15,16], multimorbidity management [17,18] dementia diagnosis and treatment [10,19], palliative care [14], oral health in older patients [9] and the use of geriatric assessment [20,21]. However, to our knowledge, no studies have addressed GPs’ perspectives regarding the current situation and rising challenges of ambulatory geriatric care in general.

Hence, we were interested in understanding how the issue of primary care for older people with complex health problems is conceived of by general practitioners. We chose qualitative methods using semi-structured interviews to explore this area. The leading research questions were ‘How do general practitioners view their role in primary care for older people with complex health problems?’ and ‘What lines of action do they subsequently take?’. We hypothesized that geriatric training has a major impact on GPs’ views.

## Methods

The study was nested in the project OMAHA II Maintaining autonomy among community dwelling vulnerable elders by individualized case management in primary care, a non-randomized controlled intervention study on the effects of individualized case management for general practice patients aged 70 and older in Berlin. The study was conducted jointly by the Charité Universitätsmedizin Berlin and the Robert Koch Institute Berlin from 2011 to 2013. The study was conducted as a part of the Research Collaboration Autonomy Despite Multimorbidity in Old Age: Interventions to Mobilize Resources (AMA II) in Berlin, Germany.

The main study was performed with the approval of the ethics committee of the Charité Universitätsmedizin Berlin. For the research reported in this paper, we assured informed consent of the interviewees and data protection in accordance with Charité’s official data protection officer. We report the results in accordance with the COREQ guidelines [22].

Due to limited resources for the qualitative within the main study we aimed to recruit a sample of ten interview participants and excluded theoretical sampling and data saturation as aims for the sampling process. Potential interview participants had to be actively working in office practices or primary healthcare centers. We approached eligible GPs who were either cooperating in the OMAHA II intervention study or had previously been involved in general practice research projects. As we were in contact with 20 eligible interview participants, we could sample on the basis of categories and include GPs with single and shared practices in three German cities with different degrees of geriatric qualification, which was in our interest due to the initial hypothesis of its possible impact on different views. The geriatric qualifications were defined as follows: (a) *formal* in persons with a formal qualification as a geriatrician or complementary training in geriatrics for GPs; (b) *non formal* for geriatric expertise that was not formally acquired but was non-formally acquired through working experience in a geriatric institution; and (c) *none* where neither of these criteria were met. Interviewees were contacted by postal mail. All of the professionals approached agreed to be interviewed on the basis of information about the study aim and data protection policy. No reimbursement or gratification was granted.

Data were collected by semi-structured expert interviews in October and November, 2011. Three were conducted by telephone, and seven were conducted in person. All interviews were conducted by the same experienced interviewer, who was employed part-time as a research fellow within the project and had been trained in qualitative interview techniques during her M.A. in educational sciences and in previous qualitative studies. She had no contact with any of the interview participants before recruiting them and presented herself to them as a social scientist with experience in gerontology but a layperson concerning medical issues. The duration of interviews was 30 to 90 minutes. All of the interviews were tape recorded and fully transcribed. In addition to the recordings, notes concerning the atmosphere as perceived by the interviewer were taken. Where necessary, notes about statements that were not recorded because the interview topic was recommenced unexpectedly after a first official ending and goodbye were recorded. No repeat interviews were carried out. The transcripts were not returned to the GPs for comment or correction.

An interview guide was pilot tested with one general practitioner from the study team in advance. It requested the GPs give a general overview of their practice and describe the prevalence of older people with complex health problems and importance of geriatric disease patterns in their daily work, which services they usually offer to older people with complex health problems, and what challenges they encounter in this context. Additionally, it contained a list of geriatric assessment instruments and a sample-collection of graphic presentations of results, and GPs were asked about their experiences and preferences with different items. The answers to these questions, however, have mainly been used as inputs for the development of a geriatric screening instrument and are not subject to the study at hand. Additionally, professional background (specializations, professional experience) was ascertained.

We aimed to elaborate on the characteristic views of GPs on primary care for older people with complex health problems. GPs’ perspectives were conceptualized to be social representations, i.e., systems of values, ideas, and action patterns shared within groups concerning a certain subject - in this case, primary care for older people with complex health problems. Social representations are cultural frameworks that serve as orientation to the individual and enable intra-group communication [23,24]. Through this emphasis, the concept differs from other possible theoretical frames, e.g., subjective theories, and we chose this socio-psychological angle because within the OMAHA project as an intervention study, we were interested in understanding how the implementation of interventions in primary care could best be designed to fit how GPs oriented their actions. So, during analysis, the leading viewpoint was ‘which values, ideas and action patterns appear to serve as orientations?’ Analysis was carried out by the researcher who also conducted the interviews, using MS word and started with open coding on a case basis and case-descriptions. The emerging thematic structure was then repeatedly discussed within the whole study group (the authors, all of whom read all interviews). Initial ideas for categories were explored and often times dismissed (e.g., ‘image of aging’ did not prove to be distinctive within our text corpus), using paradigmatic examples and the explicit search for contrasting examples. The categories carved out as substantial (role definition, definition of professional domain, and action pattern with regard to cooperation) were enriched with comparative dimensions through reiterated inter-case comparison and developed into an empirically grounded multi-dimensional typology [25].

We succeeded in including 6 female and 4 male GPs with specializations in general and internal medicine, working in different practice forms in the sample. Two GPs had a single practice, four GPs were engaged in practice sharing, and four GPs worked in an ambulatory healthcare center. Three of the GPs had formal, one had non-formal, and six had no geriatric qualification.

## Results

### Three types of views

We identified groups of GPs with different types of representation of primary care for older people with complex health problems: (A) ‘maneuvering along competence limits’, (B) ‘Herculean task’, and (C) ‘cooperation and networking’ (Table 1). Three dimensions were identified to characterize and compare the respective types: (1) GPs’ perception of their own role, (2) GPs’ definition of their professional domain, and (3) GPs’ patterns of interaction with other professions and institutions. Three of the four GPs with non-formal or formal geriatric qualification pertain to type (C); that is, all GPs in this group have non-formal or formal geriatric qualification. Group (B) comprised one GP with formal and one with no geriatric qualification, and group (A) comprised five GPs with no geriatric qualification.

**Table 1:** **Three types of views**

**Dimension**	**(A) ‘maneuvering along competence limits’**	**(B) ‘Herculean task’**	**(C) ‘cooperation and networking’**
(1) GPs' perception of their own role	Solitary medicine expert	Companion through life	Cross-linked medicine expert
(2) GPs' definition of their professional domain	Narrow, fragmented	Wide, holistic	Wide, holistic
(3) GPs' action patterns in primary care for older people with complex health problems	Physician-centered (restricted)	Physician-centered (expansive)	Cooperative, cross-linking

#### (A) ‘Maneuvering along competence limits’

The representatives of this type perceive primary care for older people with complex health problems to be a challenge confronting them with the limits of their mandate and capacities, such as uncertainties resulting from the challenges of being a facilitator between patients and specialists or experiences of helplessness when being confronted with patients’ social or care-related shortcomings. However, most examples address a lack of time for consultation and treatment and lacking prescription-budgets.

##### Role definition in (A)

GPs in this group see themselves as stand-alone medical experts and service providers. Second, they share a rather technical understanding of their work.

‘And these ideas, yes, that the doctor assumes quasi pastoral function - of course many elderly people have that. And, I feel honored when I'm credited with that. Have to say, though, that I haven’t been trained for it; I am not qualified in any special way. However, there are simply people who just want technical service from me. Remove the pain. Why they have knee-pains or so on, they do not at all want me to assess such things’. (OMAHA-II-2011-t-04, 336 ff).^b^

Some physicians in the group explicitly identify themselves as ‘entrepreneurs’. Most, however, take a merely pragmatic or even critical stance toward the economic frame binding them.

‘If that were to be part of my tasks, it has to be linked to my budget. I don’t want extra money for this; but still, I want to be able to do something in this respect so that it doesn’t simply run contrary to economic practice management. And, economically reasonable practice management isn’t that which is best for the people, but it is so that I don’t go broke with my enterprise’. (OMAHA-II-2011-t-04, 812 ff).

##### Definition of professional domain in (A)

GPs in this group define their professional domain in close fit to formal resources and requirements, mainly defined by their training and remuneration by the German health insurance.

‘At the moment I have lots of women to whom their wartime experiences come to mind again […] and who start to talk of these. I could spend hours listening. They would surely appreciate such attention. However, health insurance would not appreciate paying for this. And again, I don't believe I am trained for this. I am trained to prescribe pills’. (OMAHA-II-2011-t-04, 450 ff).

A sense of injustice is often expressed about being blamed for not providing comprehensive care:

‘We’re not that bad, but we can do only what we have the means for. We’re not too stupid to recognize when people have a need for rehab or training or anything else. Only, it is no use that we recognize it if we can’t do anything then’. (OMAHA-II-2011-t-04, 131 ff).

##### Action pattern in (A)

The dyadic doctor-patient interaction is the paradigmatic situation in the narrations in type (A), with the GPs being supported by their practice team. Worries exist about conflicts of interest with professionals external to the practice, e.g., in the following quote, a GP explains why he would prefer a case manager^c^ within the practice team over an external one:

‘It’s as if we are made of one piece, and she will have a certain - I also say that, that plays a role as well – loyalty towards me. Yes? More than, I may say, a stranger who comes there and says ‘Well what? The doctor didn’t prescribe walking training?’ My assistant would never say such a thing because she knows that I can’t prescribe it because my budget for physiotherapy is limited’. (OMAHA-II-2011-04, 228 ff).

##### Strategies for dealing with competence limits in (A)

We find three different strategies for dealing with competence limits in the group. The first is to define the limits of legitimate requests quite narrowly and reject demands to go beyond. An extreme example is one physician’s opinion that from the moment a person is in a state of frailty, they are no longer the usual patient of a GP, assuming that the person will almost necessarily move into a nursing home and that the ‘normal’ GP is no longer responsible from that time on. Other GPs in the group, however, utter frustration.

[Through geriatric assessment] ‘Yes, well, we are merely picturing the misery, and then we can’t do anything’. (OMAHA-II-2011-04, 10 ff).

The second strategy is budgeting resources. It ranges from the perfection of office management to rather precarious but permanent workarounds for structural shortcomings, such as redistributing time resources:

‘If you’ve got as many young and healthy patients as possible who only come with a cold occasionally, then you can work up a bolster allowing you to speak longer with the others – that’s how it basically is’. (OMAHA-II-2011-p-09, 64 ff).

The third strategy is long-term involvement in supporting change processes, e.g., participating in studies, doing research on the development of instruments and practices, or maintained active membership in professional and scientific associations.

#### (B) Herculean task

Primary care for older people with complex health problems means comprehensive dedication to the whole of patient well-being by the GPs in group (B). It requires the use of a wide spectrum of means including rather unorthodox ones and altogether takes on a Herculean extent.

##### Role definition in (B)

The GPs in this group see themselves as companions through the life course.

‘You are the patient’s advocate, and you care for all areas of life, considering them in what you do next’. (OMAHA-II-2011-p-17, 589 ff).

It is essential for the fulfilment of their role to be both professionally and personally involved and committed beyond the usual.

‘I dedicate so much time here. […] I make home visits practically every day before consultation hours, and now, how does the rest of the day look? Well, when we’ll be finished now [around six o’clock on a Monday evening], I drive to a home visit first. Then, I drive to a clinic and then to a nursing home. Then, I drive to the home of that patient in the wheelchair. Young man. Then, I go for another home visit, and then I go home and see to it that I write another report. That’s it’. (OMAHA-II-2011-p-17, 571 ff).

Close familiarity with the patients, as well as their social and local surroundings, are seen to be essential.

‘I brief the savings bank and they will call me and say ‘This cannot be happening, she wanted to draw 25.000’Mark’ [Note: ‘Deutsche Mark’ was the German currency before the country entered the Euro-zone.]. […] Well, you have to try and put up nets, otherwise they get lost. And I try and explain this to the bakery as well: ‘In case something strikes you as odd, call me!’ (OMAHA-II-2011-t-11, 485 ff).

##### Definition of professional domain in (B)

These GPs define their professional domain widely and holistically. The comprehensive health- and well-being of their patients is their scope of action, and they adopt a perspective that draws very much on the individuality of each person and situation.

‘You can’t describe a disease in numbers. Because everyone reacts differently to certain things of course. One responds more to attention, the care-related pitfalls, empathy for his suffering. One says ‘I am entitled to this, I’ve paid my ten Euros^a^ after all, I want the full program of medication, all there is!” (OMAHA-II-2011-p-17, 36 ff).

##### Action pattern in (B)

Quite similarly to the understanding in type (A), the interaction pattern in (B) is physician-centered. In contrast to that of the first group, however, the action pattern is also extended widely beyond what is formally required.

‘Well, I actually have gone as far as to not just make a home visit with older folks but sometimes also to go shopping for them. Yes, I get going and bring some food so they would have something in the fridge once in a while’. (OMAHA-II-2011-p-17, 188 ff).

The explicit reason for acting in this way is the great need encountered with patients in precarious situations, even at high costs to the GPs themselves. The following quote concerns a situation where a patient was found incapable of remaining alone in the proper housing.

‘I employ medical assistants. In a case like this, they will sit and phone the insurance and the nursing home. That is actually not their job, and I pay. The correct way would have been for me to get the patient to the hospital with some constructive diagnosis – wouldn’t have been in her interest, especially as a geriatric patient’. (OMAHA-II-2011-t-10a, 125 ff).

##### Opposition toward economic motivation and bureaucratic structures in (B)

The GPs in group (B) note how their efforts are about responding to personal needs in an appropriate, namely human manner and out of an intrinsic motivation rather than for economic benefit.

‘Yes, well I am supposed to be more economic. Whether patients fall by the wayside thereby is a second question. That’s my responsibility again, not the health insurance’s. [That is] the straight statement that doctors still address people and insurances juggle numbers’. (OMAHA-II-2011-p-17, 31 ff).

The GPs prefer informal, quick structures with great closeness to the patient’s life-world.

[I wish for] ‘a less complicated social structure, where you could just call and say: ‘here, I don’t know how to go on’. Just as I said once, where I grew up, nurse Sigrid, who was simply there and went around on her bicycle’. (OMAHA-II-2011-t-10, 637 ff).

#### (C) Cooperation and networking

The GPs in this group understand the limits of their own service to the patient, not as a limit of care for older people with complex health problems but more as points where another provider takes over.

##### Role definition in (C)

The GPs in group (C) define their role quite similarly to those in group (A) as that of medical experts. However, in contrast, they see it as an essential part of their role to be cross-linked to other actors important for the care of older people with complex health problems. They understand themselves to be hubs in a network and perceive of the benefits of cooperation and networking to be professional successes.

‘We have developed a net of institutions over time, with which we work so that we can make offers according to needs. You see, there is the day-care clinic and mobile rehabilitation. We have a number of settled physiotherapists, occupational therapists, and neuropsychologists with whom we work quite well. Not to forget the coordination center so that you can actually offer something for every constellation’. (OMAHA-II-2011-p-07, 52 ff).

Interviewer: ‘Do you give counsel on advanced health care directives yourself?’

GP: ‘Seldom. Frankly I lack the time. Because that is such a complex issue and one should do it reasonably. And it is all about values and stuff, not specifically about medicine in my opinion. So I always point out that each institution you feel related to ethically, religiously, or otherwise provides information and pre-formulations according to the proper understanding of the world. […] And for the rest, if medical questions remain, I offer that one can talk it through with me again’. (OMAHA-II-2011-p-07, 102 ff).

##### Definition of professional domain in (C)

The professional domain in group (C) is as wide and holistic as in group (B) in the sense that the complex patient situation and well-being is regarded to be the proper subject matter. In contrast to group (B), however, physicians in (C) concentrate their own direct activity on medical issues and regard it to be their task to understand enough of non-medical aspects to know to whom they should pass the baton.

‘Yes, well we are very happy in the first place that we have the case manager whom we can send at times to do a home visit, which perhaps is not yet medically necessary, to sort out the pitfalls at home – need for assistive equipment, such stuff. And to determine whether applying for a care level may yet be sensible’. (OMAHA-II-2011-p-22, 57 ff).

##### Action pattern in (C)

The work-ratio of the GPs in group (C) is cooperative. Using and building institutional networks is distinctive for these GPs. Referrals or making contact appear to be services in their own right.

‘Sometimes, you can do simple things. To make the connection with the church parish again when someone says ‘spiritual life is so important to me and somehow I don’t get in contact anymore”. (OMAHA-II-2011-p-07, 572 ff).

##### Expectations and reflection on cooperation in (C)

GPs in group (C) share rather positive expectations toward the benefits of cooperation and networking and a higher trust in other actors’ impact compared to the two other groups, acknowledging also a possible transfer of expertise.

‘I notice increasingly that it is a curious thing that the care sector is much more advanced in these assessment-areas than medics are. […] There’s more circulating than you would expect as a doctor and perhaps it were reasonable to take up one or two things, yes, into everyday practice’. (OMAHA-II-2011-p-20, 130 ff).

Yet, they also reflect on challenges and prerequisites for good cooperation.

‘Especially in the case of dementia, it is obvious that the caregivers – professionals or family members – have the decisive role in the communication with the patient. […] So, as a GP, you depend much on being informed well and knowledgeably. And whether that occurs is very different’. (OMAHA-II-2011-p-20, 168 ff).

In addition to such barriers, the GPs in the group report that the degree to which they succeeded in building cooperation was limited by structural conditions. Only one of the three was, at the time of the study, in a position to rely on such a network as she should like. She was the only one to regard her practice as one with a geriatric focus. This included offering an extensive physician-conducted geriatric assessment as a regular service. The other two rather observed a gap between aspiration and reality – according to their own evaluations, clearly due to budget restrictions.

#### A shared complaint: Insufficient remuneration

Remuneration modalities appear in all of the interviews as structuring conditions for GP activities and decisions, even if the GPs take different stances toward them as we have shown above.

‘All is determined by the remuneration modalities, and they mean you get a lump-sum system. Therefore, older people with complex health problems – in case they really are geriatric and not simply old – are highly loss-making. For much effort, much explaining, much visiting. And you’ve only got two positions to bill and that’s it and they don’t become more no matter how much you talk. Insofar it isn’t good financially’. (OMAHA-II-2011-p-09,57 ff).

‘All this being the patients‘advocate and caring for all areas of life and involving them in what you do is utterly uninteresting to the health insurance companies but is interesting for only doctors and patients. However, naturally, it is not being outright supported. Well, you could achieve much more with much less. However, that isn’t wanted, narrative medicine’. (OMAHA-II-2011-p-17,589 ff).

Interviewer: Does it pay off, financially, to conduct it [the geriatric assessment as scheduled in the German health insurances’ catalogue of refundable treatments]?’

‘Not really. If you think that it takes thirty to forty minutes per person, if you want to do it reasonably, not to speak of all the following work, then it is of course feasible only to a limited degree’. (OMAHA-II-2011-p-07,151 ff).

## Discussion

### Summary of results

We asked ‘How do GPs view their professional mandate and capacities in the provision of comprehensive care for older people with complex health problems?’, and we found three types of views within the sample. They are distinguished by different perceptions of the GPs of their own role, the professional domain, and action patterns.

The typology supports the initial hypothesis that geriatric expertise has a major impact on how GPs perceive of primary care for older people with complex health problems and their own tasks in it.

According to our conceptual framework of social representations as orientations of action and from the self-reports of the GPs, we may assume that different representations result in different treatment of older people with complex health problems and ultimately different qualities of care. Focus on single diseases rather than complex situations may produce adverse outcomes [26]. Thus, the narrow definition of the professional domain may be a disadvantage for patients.

Insufficient remuneration modalities are seen to be a limiting condition by GPs from all groups and can thus be seen as a major barrier for the implementation of changes in primary care for older people with complex health problems.

A barrier specific to type (A) is the phenomenon of declining tasks that are tied to comprehensive geriatric care which we have presented under the title of coping with competence limits. In one perspective, we can see this to be a causal loop or the self-stabilizing nature of blind spots. For example, a lack of interest in specialized services offered by non-physician health care professionals increases a lack of knowledge about such services, which in turn makes the option of cooperation even less interesting. From another perspective suggested by our interviews, the declination of tasks is the result of an effective and highly needed strategy of self-protection against overburdening and frustration on the side of the GPs. Examples of GPs in group (B), with their high self-expectations and commitment, may well foster the concern in their colleagues that embracing the tasks of comprehensive approaches to primary care for older people with complex health problems is likely to lead straight to definite over-burdening.

We hypothesized that geriatric training or experience might have a major impact on the ways in which GPs view their mandate and capacity in caring for older people with complex health problems. Now, it will already have occurred to those readers who are familiar with geriatric medicine that the definitions of role, domain and action pattern of type (C) come closer to the picture of a geriatrician than those of the other types. This was our impression when we finished the typology, and in the following, we will underpin this by drawing on the 2004 position statement of the European Union Geriatric Medicine Society (EUGMS) [5]. We extracted the following quotes as key-statements on the subjects of general role definition, definition of the professional domain, and action patterns, i.e., the dimensions of the typology:

‘A geriatrician combines obtaining a sound medical and social history with the ability to comprehensively assess and examine older patients’. (p.192)

He ‘directs and advises a multidisciplinary team’. (p.191)

So, according to EUGMS, the general role of a geriatrician is that of the specialist in geriatric medicine who leads a multidisciplinary team.

‘Geriatric medicine can be described as ‘the specialty for health-related problems in older people […]. The term ‘health-related problems’ emphasizes the interaction between physical, mental, emotional, social and environmental aspects.’ (p. 191) ‘The geriatrician has knowledge of palliative care, of health promotion and preventative health care and of the local social support system’. (p.191)

In the context of our typology, this should clearly be summarized as a wide and holistic definition of the professional domain.

‘The EUGMS is well aware of the need for co-operation with other hospital and community specialties, both for training and the implementation of new technology and ideas’. (p.192)

Thus, we can speak of an action pattern of cooperation and cross-linking as the proclaimed norm. So, we see in Table 2 that our type (C) coincides in two dimensions with the picture of the geriatrician as drawn by the EUGMS statement.

**Table 2:** **Comparison with ideal-typical geriatrician**

**Dimension**	**(A) ‘maneuvering along competence limits’**	**(B) ‘Herculean task’**	**(C) ‘cooperation and networking’**	**Geriatrician according to EUGMS**
(1) GPs' perception of their own role	Solitary medicine expert	Companion through life	Cross-linked medicine expert	Geriatric specialist, leader of a multidisciplinary team
(2) GPs' definition of their professional domain	Narrow, fragmented	Wide, holistic	Wide, holistic	Wide, holistic
(3) GPs' action patterns in primary care for older people with complex health problems	Physician-centered (restricted)	Physician-centered (expansive)	Cooperative, cross-linking	Cooperative, cross-linking

All of the members of group (C) are trained and/or experienced in geriatrics and refer to geriatric knowledge and standards in their narrations. Particularly, one of these GPs reflects about how the training in geriatrics changed the way of dealing with patients. We assume that the training is indeed an important factor in inducing a paradigm of cooperative working and fosters a professional self-image, such as in the type (C) group, and do not assume that the GPs in the group have had an altogether different understanding of their role and tasks in the first place. However, there was one case of one GP who is a fully trained geriatrician and yet holds rather strong reservations against institutional cooperation. Instead, we find the typical mindset of group (B) in this case. There is no necessary step from undergoing geriatric training to developing a certain mindset. Drawing on the individual case analysis, we can find an actual and/or perceived lack of essentially helpful or only competent institutional cooperation partners in the surrounding of this GP’s practice as a factor thwarting the options for establishing networks. The case could albeit also point to the possibly strong impact of role definitions once they are established.

### Comparisons with the existing literature

The psychological importance of professional self-images of GPs for developments in the whole area of primary care for older people with complex health problems has been stated before [27]. Our study supports the finding that definitions of their own role (whether conscious or not) serve to justify the exclusion and inclusion of certain sets of services. In turn, the belief that the role definition is ‘the norm’ in the sense of ‘what ought to be’ is stabilized by the fact that the exclusion of certain services is common over a certain time. Viewpoints and practices unfold self-stabilizing dynamics, immunizing against the demand for change, and have been described as ‘causal loops’ in the establishment of conventions among GPs and in patients [28].

A phenomenon of excluding certain tasks similar to the one we described for type (A) is found by Melchinger and Machleidt [19] in their study on dementia-care by German GPs. They also analyze this as a strategy in coping with competence deficits and cite justifications analogous to our own findings: skepticism toward the benefit of therapeutic efforts and an unawareness of specialized services offered by other health care professionals. The alternative interpretation of declination of tasks as a means of self-protection against overburden is in line with the risk of (re) stylizing the GP as a mythical ‘über-doctor’ who is the diagnostic filter before specialist treatment, gatekeeper to any further care, and primary reference person for any life world questions [27].

There are earlier findings that the mode of remuneration is a primary influence on GP behavior [28], in line with the demand for the development of adequate compensation systems for comprehensive, patient-centered primary care [29], including performance metrics that allow the reward of quality care [26]. The need for this has been emphasized especially for aging populations with complex care needs and the need for specialized services such as geriatric or palliative care [30-32].

### Strengths and limitations of the study

It is the strength of this study to have illuminated GPs’ views on the delivery of primary care for older patients with complex health problems in a way that allows for the understanding of barriers and chances for the implementation of changes in this area.

There are some limitations regarding our sample, however. The size of the groups, though common for a qualitative study, cannot serve as a basis for estimations about the distribution of the types among GPs in Germany. For example, it may well be that there are many more GPs with a very high degree of commitment such as in the ‘Herculean task’ group [30]. There might also be additional types to be found. Additionally, our analysis is partly focused on differences in relation to the degree of geriatric qualification. However, because there is only one GP with non-formal geriatric qualification, we have no hint to the possible impact the way in which geriatric qualification is acquired has on the resulting perspective. Finally, our analysis does not integrate such potential influential factors as age, gender, years of professional experience as a GP, or location of practice (city or rural area) [14].

Also, within our study we do not have independent data to assess the process and outcome of care quality provided by the GPs in the sample.

### Recommendations for education, research, and policy

The findings imply that geriatric training is one important factor for enhancing the contribution of GPs to multidimensional and multi-professional comprehensive care for older people with complex health problems. Therefore, the implementation of GP-targeted advanced training is a promising basis for positive development. The definition of GPs’ own role and tasks should be addressed explicitly as a part of professional training. Among the interviewed GPs, we find a partial declination of tasks related to the provision of comprehensive geriatric care and analyze it as a strategy of self-protection – mainly against overburdening. This supports the idea that changes in primary care for older people with complex health problems can be promoted by making it possible to provide comprehensive outpatient care without increasing the workload.

Further studies should investigate, however, more individual factors in the formation of GPs’ understandings of their roles, tasks, and action patterns, and deepen the understanding of education and other influences on it.

Also, the impact of different sets of GPs’ definitions of their own role, of tasks and modes of action on actual behavior and quality of care needs to be examined in future research studies applying quantitative methods and assessing objective criteria (such as mortality or declines in health status) as well as patient-centered subjective criteria (such as quality of life, or overall wellbeing).

Apart from the need to raise awareness among GPs regarding cooperation in general, however, the health care system itself should also establish and strengthen such structures. For example, more recent advancements in German legislation such as Case Management and ‘care mentoring’ (German ‘Pflegeberatung’), center their attention directly on individual patient’s needs and wants and provide active assistance and counselling regarding health care and care-related alternatives (cf. §§ 7a and 92b Sozialgesetzbuch Elftes Buch/Code of Social Law XI). Rather than installing parallel structures offering support to older patients, resources should be used to strengthen an interprofessional primary care network. Incentives for cooperation and networking could possibly help foster delegation of care across professions, thereby easing the burden on single GPs. On a more basic economic level, the more complex patient setup in geriatrics needs to be respected: the reimbursement-structure should account for the fact that more time is essential for adequate examination and medical history taking in older patients.

## Conclusions

This qualitative interview-study of ten German GPs unearths three different ways GPs view outpatient care for older people with complex health problems, identifies barriers for the development of comprehensive care in general practice and suggests possible strategies for improving primary care for older people with complex health problems.

In combination with specific definitions of their own professional role, the professional domain, and action patterns regarding cooperation and networking, the provision of primary care for older people with complex health problems is perceived to be ‘maneuvering along competence limits’ (Type (A)), a ‘Herculean task’ (Type (B)), or a task of ‘cooperation and networking’ (Type (C)).

Limited financial resources, lack of cooperational networks, and attitudes appear to be barriers to assuring better primary care for older people with complex health problems. To overcome these barriers, geriatric training is likely to have a positive impact but needs to be supplemented by regulations regarding reimbursement. Within these frameworks, finding a realistic perspective and manageable understanding of one’s role appears to be the future core challenge on the side of GPs in the primary care of older people with complex health problems.

## Endnotes

^a^By the time of the interview, German patients were required to pay a practice fee of ten Euros per quarter at the first visit in the time period.

^b^The citations allow the reader to attribute quotes to the different interview partners: All citations start with “OMAHA-II-2011-“indicating the name of the study and the year, followed by the letter ‘t’ or ‘p’ for ‘telephone interview’ or ‘personal interview’, and then the number assigned to the individual interview partner, e.g. ‘-04’.

^c^‘Case manager’ here refers to a person qualified to support individuals’ and families’ comprehensive health needs through a structured process of assessment, planning, facilitation, care coordination, evaluation, and advocacy for options and services. The issue came up during the interview because the interview partner knew that the main intervention of the OMAHA II study would be case management for patients of general practices, delivered by a case manager external to the practices.

## Abbreviations

GP: General practitioner
EUGMS: European Union Geriatric Medicine Society
